# Cruciferous vegetable intake is inversely associated with extensive abdominal aortic calcification in elderly women: a cross-sectional study

**DOI:** 10.1017/S0007114520002706

**Published:** 2021-02-14

**Authors:** Lauren C. Blekkenhorst, Marc Sim, Simone Radavelli-Bagatini, Nicola P. Bondonno, Catherine P. Bondonno, Amanda Devine, John T. Schousboe, Wai H. Lim, Douglas P. Kiel, Richard J. Woodman, Jonathan M. Hodgson, Richard L. Prince, Joshua R. Lewis

**Affiliations:** 1 School of Medical and Health Sciences, Edith Cowan University, Joondalup, WA 6027, Australia; 2 Medical School, The University of Western Australia, Perth, WA 6000, Australia; 3 Park Nicollet Osteoporosis Centre and HealthPartners Institute, HealthPartners, Minneapolis, MN 55416, USA; 4 Division of Health Policy and Management, University of Minnesota, Minneapolis, MN 55455, USA; 5 Hinda and Arthur Marcus Institute for Aging Research, Hebrew SeniorLife, Department of Medicine, Beth Israel Deaconess Medical Center, Harvard Medical School, Boston, MA 02215, USA; 6 Flinders Centre for Epidemiology and Biostatistics, Flinders University, Adelaide, SA 5042, Australia; 7 Centre for Kidney Research, Children’s Hospital at Westmead School of Public Health, Sydney Medical School, The University of Sydney, Sydney, NSW 2006, Australia

**Keywords:** Abdominal aortic calcification, Vascular calcification, Arteriosclerosis, Atherosclerosis, Cruciferous vegetables, Vegetables, Women

## Abstract

We have previously shown that higher intake of cruciferous vegetables is inversely associated with carotid artery intima-media thickness. To further test the hypothesis that an increased consumption of cruciferous vegetables is associated with reduced indicators of structural vascular disease in other areas of the vascular tree, we aimed to investigate the cross-sectional association between cruciferous vegetable intake and extensive calcification in the abdominal aorta. Dietary intake was assessed, using a FFQ, in 684 older women from the Calcium Intake Fracture Outcome Study. Cruciferous vegetables included cabbage, Brussels sprouts, cauliflower and broccoli. Abdominal aortic calcification (AAC) was scored using the Kauppila AAC24 scale on dual-energy X-ray absorptiometry lateral spine images and was categorised as ‘not extensive’ (0–5) or ‘extensive’ (≥6). Mean age was 74·9 (sd 2·6) years, median cruciferous vegetable intake was 28·2 (interquartile range 15·0–44·7) g/d and 128/684 (18·7 %) women had extensive AAC scores. Those with higher intakes of cruciferous vegetables (>44·6 g/d) were associated with a 46 % lower odds of having extensive AAC in comparison with those with lower intakes (<15·0 g/d) after adjustment for lifestyle, dietary and CVD risk factors (OR_Q4 *v.* Q1_ 0·54, 95 % CI 0·30, 0·97, *P* = 0·036). Total vegetable intake and each of the other vegetable types were not related to extensive AAC (*P* > 0·05 for all). This study strengthens the hypothesis that higher intake of cruciferous vegetables may protect against vascular calcification.

CVD continues as the leading cause of death globally^(1,2)^. The major underlying cause of CVD is atherosclerosis, the progressive accumulation of fatty deposits, inflammatory cells, Ca and other substances within the intimal layer of the arterial walls^(3)^. Calcification of the arteries involves Ca accumulation within the intimal or medial layers of the arterial walls. Intimal calcification is considered as an indicator for advanced atherosclerotic plaques^(4)^. However, it is now understood that calcification of the vasculature is a highly regulated process that can precede or occur independently of atherosclerotic lesions^(4)^.

Abdominal aortic calcification (AAC) can be classified as a marker of subclinical atherosclerosis and has been shown to predict CVD outcomes^(5,6)^. Calcification of the abdominal aortic and iliac artery walls is visible when imaging the thoracic and lumbar spine to assess the presence of vertebral fractures. The assessment of calcification can easily be incorporated at the time of routine bone density screening in older adults^(6)^. The extent of calcification can be scored using the Kauppila AAC 24 scale (AAC24)^(7)^. This scoring system has been shown to have good-to-very good agreement using standard radiographs^(8,9)^. We have also previously shown that extensive AAC using the cut point of AAC24 ≥ 6 is associated with an 80 % higher risk of having an atherosclerotic-related death compared with individuals with lower scores^(6)^.

An unhealthy diet can substantially increase an individual’s risk for developing CVD^(10)^. Improving one’s diet is a simple and cost-effective strategy that can substantially reduce the individual and societal burden of CVD. A high intake of vegetables is one of the cornerstones for a healthy diet and is consistently associated with a reduced risk of CVD^(11)^. However, different vegetables contain different proportions of bioactive phytochemicals^(12)^. Thus, it is likely that not all vegetables are the same in terms of their protective effects and pathways involved^(11)^.

We have previously shown that a higher intake of cruciferous vegetables is associated with lower carotid artery intima-media thickness^(13)^, a marker of atherosclerosis, and a lower risk of atherosclerotic vascular disease mortality^(14)^. To further test the hypothesis that an increased consumption of cruciferous vegetables protects against CVD, we aimed to investigate the cross-sectional association between cruciferous vegetable intake and extensive calcification in the abdominal aorta. We hypothesised that higher intake of cruciferous vegetables will be associated with a lower odds of having extensive AAC.

## Materials and methods

### Ethical statement

Written informed consent was obtained from all participants, and the Human Research Ethics Committee at the University of Western Australia approved the study.

### Study population

Participants included were originally recruited to a randomised controlled trial of Ca supplementation to prevent osteoporotic fractures in older women. This randomised controlled trial, the Calcium Intake Fracture Outcome Study, has been described elsewhere^(15)^. Briefly, 1500 women were randomly assigned to receive either daily 1·2 g/d calcium carbonate or a matching placebo. In a sub-study, thirty-nine participants received 1·2 g/d calcium carbonate plus 1000 IU (25 μg) of vitamin D_2_ daily^(16)^.

The participants were recruited in 1998 from the Western Australian general population by using the electoral roll and randomly selecting women aged 70 years and above to receive mailed invitations. Registration on the electoral roll is a requirement of citizenship in Australia. Of the 5586 who responded to the invitation, 1500 women were eligible and were recruited into the study. All women were ambulant and expected to survive beyond 5 years. Receiving medication known to affect bone metabolism, including hormone replacement therapy, was an exclusion criterion. The women randomised into the Calcium Intake Fracture Outcome Study were more likely to come from higher socio-economic groups compared with the general population of the same age.

Of the 1500 participants, we excluded those with missing AAC data (*n* 417), missing dietary data (*n* 5), implausible energy intakes (<2100 kJ (500 kcal) or >14 700 kJ (3500 kcal))^(17,18)^ (*n* 9), those with previous clinical diagnosis of atherosclerotic-related disease (IHD; heart failure; cerebrovascular disease, excluding haemorrhage; and peripheral arterial disease) (*n* 117) and diabetes mellitus (*n* 48) and those with missing data on covariates (*n* 220). Previous clinical diagnosis of atherosclerotic-related disease and diabetes mellitus was *a priori* exclusion criteria for the current analyses, as clinical diagnosis may have resulted in dietary changes thereby attenuating the outcomes of interest. Methodology of the assessment of prevalent atherosclerotic-related disease and diabetes mellitus has been described elsewhere^(19)^.

### Abdominal aortic calcification assessment

All study participants had a digitally enhanced single-energy image of the thoracolumbar spine captured using a Hologic 4500A densitometer (Hologic). Images were collected in 1998 and 1999. An experienced investigator (J. T. S.) scored each image for AAC using an established technique^(5,8,20)^. Both intra- and inter-rater agreements by J. T. S. have been reported as very good^(7,20)^. The anterior and posterior aortic walls were divided into four segments. Each segment corresponded to an area in front of the lumbar vertebrae (L1–L4). Aortic calcification was scored: 0 (no calcification of the aortic wall), 1 (≤1/3 calcification of the aortic wall), 2 (>1/3 to ≤2/3 calcification of the aortic wall) and 3 (>2/3 calcification of the aortic wall). Scores ranged from 0 to 6 for each segment, and therefore total scores ranged from 0 to 24^(5,7)^. More than 99·5 % of the images were of sufficient quality to assess AAC^(6)^. AAC scores were then re-categorised as not extensive (AAC scores 0–5) and extensive (AAC scores 6–24), as previously used investigating the aetiology of AAC^(6,21,22)^.

### Dietary intake assessment

Dietary intake was assessed in 1998 using a self-administered semi-quantitative FFQ^(23–25)^. The FFQ measures usual frequency of food intake for the previous 12 months and comprises a list of seventy-four foods with ten frequency response options ranging from ‘never’ to ‘three or more times per d’. It was complemented by another twenty-seven food and alcoholic beverage items that ask various questions, such as ‘What type of milk do you usually use?’. Portion size was calculated using three photographs of scaled portions for four different food types. Energy and nutrient intakes were estimated by the Cancer Council of Victoria using the NUTTAB95 food nutrient database^(26)^ and were supplemented by other data where necessary. Food items (including twenty-five vegetable items) were individually calculated by the Cancer Council of Victoria in g/d. Although total vegetable intake has not been specifically validated for the FFQ used in this study, particular nutrients that can be classified as markers of vegetable intake have been shown to have reasonably good agreement in a previous validation study^(24)^. For example, the energy-adjusted log values for Pearson correlation coefficients for *β*-carotene, fibre and vitamin C all had reasonably good agreement between weighed food records and the FFQ used in our study (*r* 0·43, 0·66 and 0·52, respectively). Furthermore, as the frequency component of FFQ tends to overestimate vegetable intakes, the Cancer Council of Victoria designed the FFQ to include an adjustment^(24)^. This adjustment included an additional question of how many different vegetables participants consumed on a given day to scale up/down intake of vegetables from the frequency data obtained. In addition to this, an image was used specifically for vegetables to estimate portion size. Therefore, we have confidence that the FFQ data give a good estimate of total vegetables and the classified types of vegetables, as previously published^(13,14,27,28)^.

Total vegetable intake was calculated per serving (75 g/d) according to the Australian Dietary Guidelines^(29)^. ‘Potatoes, roasted or fried, including hot chips’ were not included in the total amount of vegetables as hot chips are not recommended as part of a healthy diet^(29)^. ‘Potatoes cooked without fat’ were included. Vegetables were grouped based on the 2013 Australian Dietary Guidelines^(29)^ and were modified slightly according to specific bioactive compounds of interest. Vegetable types were: cruciferous vegetables (cabbage, Brussels sprouts, cauliflower and broccoli) – sources of organosulphur and polyphenolic compounds, such as isothiocyanates^(30)^ and flavonols^(12)^; allium vegetables (onion, leek and garlic) – sources of organosulphur compounds, such as cysteine sulphoxides and gamma-glutamylcysteines^(30)^; yellow/orange/red vegetables (tomato, capsicum, carrot and pumpkin) – sources of carotenoids, such as lycopene and *β*-carotene^(31)^; leafy green vegetables (lettuce and other salad greens, celery, silverbeet and spinach) – source of nitrate^(32)^; and legumes (peas, green beans, bean sprouts and alfalfa sprouts, baked beans, soya beans, soya bean curd and tofu and other beans) – sources of polyphenolic compounds, such as isoflavonoids and saponins^(12,33)^.

### Covariates

Age at baseline was calculated in years from date of birth until date of baseline visit. Smoking status and physical activity were obtained from a standard questionnaire. Smoking status was coded as never smoker or former/current smoker. Former/current smoker was defined as smoking >1 cigarette/d for >3 months at any time during the participants’ life. Physical activity (kJ/d) was estimated using a validated method taking into account the type of activity, time engaged in the activity and the participants’ weight^(34–36)^. The 2013 Australian Dietary Guidelines^(29)^ adherence score was used as a measure of diet quality and was calculated based on Thorpe *et al*.^(37)^. The score did not incorporate adherence to vegetable recommendations as vegetables were our exposures of interest. The components and scoring methods used to estimate the adherence score are shown in online Supplementary Table S1. Body weight (kg) and height (m) were measured using digital scales and a wall-mounted stadiometer, respectively. Participants were instructed to wear light clothing with no socks and shoes. BMI (kg/m^2^) was calculated using weight and height values. Participants provided a detailed medical history and list of prescribed medications at baseline (1998). Use of medications was verified by participants’ general practitioner, where possible. Use of antihypertensive and statin medications was used in multivariable-adjusted models to adjust for hypertension and hypercholesterolaemia, respectively. Creatinine and cystatin C were measured in baseline serum^(38)^. Creatinine was measured using an isotope dilution MS-traceable Jaffe kinetic assay for creatinine on a Hitachi 917 analyser (Roche Diagnostics GmbH). Cystatin C was measured using a fully automated particle-enhanced immunoturbidimetric assay with Sentinel Diagnostics reagents (Sentinel CH) on the Architect ci 16200 System (Abbott Laboratories) according to the manufacturer’s instructions. The estimated glomerular filtration rate was calculated using the Chronic Kidney Disease Epidemiology Collaboration creatinine and cystatin C equation^(39)^. The combined creatinine–cystatin C equation was used as this has been shown to be superior in predicting measured glomerular filtration rate compared with equations based on creatinine alone^(39)^. Due to an unhealthy lifestyle being associated with chronic kidney disease^(40)^, and individuals with chronic kidney disease demonstrating accelerated vascular calcification^(41)^, we included estimated glomerular filtration rate in our multivariable-adjusted models.

### Statistics

All data were analysed using STATA software, version 15.1 (StataCorp LP). Descriptive statistics are presented as either mean values and standard deviations, medians and interquartile ranges, or as numbers and percentages. Binary logistic regression was used to examine associations between exposures of interest and primary (extensive AAC) and secondary (presence of AAC) outcomes of interest. Primary (cruciferous vegetables) and secondary (total vegetables, leafy green vegetables, allium vegetables, yellow/orange/red vegetables and legumes) exposures of interest were entered into models as continuous variables and were also categorised into quartiles to allow for possible non-linear relationships. Linear trends across quartiles were tested using the median value within each quartile group as a continuous variable. Three models of adjustment were used: model 1 included the unadjusted model; model 2 included age, the Calcium Intake Fracture Outcome Study treatment code, smoking status, physical activity, diet quality, total energy intake and other vegetables (i.e. non-cruciferous vegetables when cruciferous vegetables were the exposure of interest) and model 3 included all variables in model 2 plus BMI, use of antihypertensive medication, use of statin medication and estimated glomerular filtration rate. Statistical significance was set at a two-sided type 1 error rate of *P* < 0·05.

#### Additional analyses

We conducted Spearman’s rho correlations between intakes of cruciferous vegetables and total vegetables, leafy green vegetables, allium vegetables, yellow/orange/red vegetables and legumes. We have previously shown that higher apple intake is associated with a lower odds of having extensive AAC^(21)^. Therefore, we used Spearman’s rho correlations to further investigate the relationship between intake of apples and cruciferous vegetables. In addition, we further adjusted our findings for apple intake using model 3.

Similar to those with a clinical diagnosis of diabetes, participants with hypertension and/or hypercholesterolaemia may have been advised to change their diet as a result of their clinical diagnosis and confound the results of our study. Given the age of the participants, a large proportion were prescribed antihypertensive medications (*n* 257/684; 37·6 %) or statin medications (*n* 99/684; 14·5 %). Excluding these participants would have resulted in an even smaller sample size (*n* 384/684; 56 %). Therefore, it was not feasible to exclude these participants. As an alternative, we explored our findings among participants prescribed medications *v*. those not prescribed medications using stratification analysis including all covariates in model 3.

## Results

### Characteristics of the study sample

**Fig. 1.**
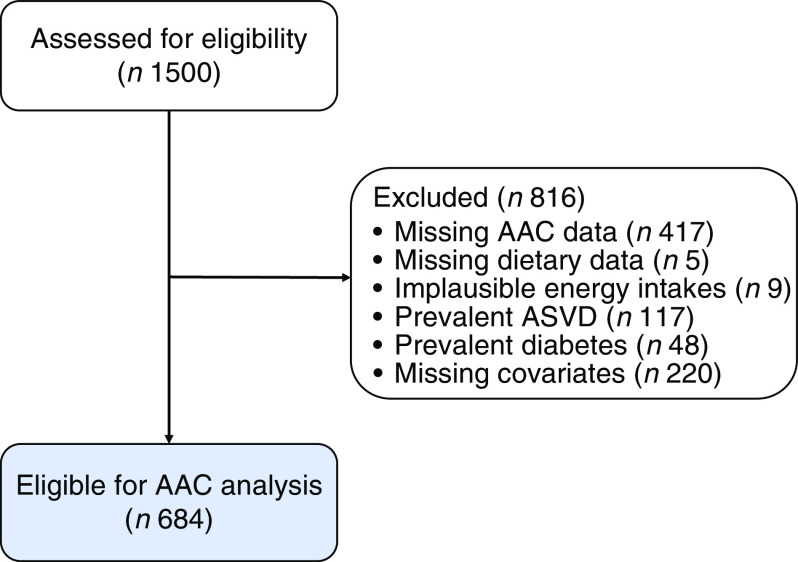
Participant flow chart. AAC, abdominal aortic calcification; ASVD, atherosclerotic vascular disease.

After pre-specified exclusions, there were 684/1500 (45·6 %) participants of the original cohort left for analysis (Fig. 1). Complete AAC data were not available for 417/1500 (27·8 %) participants as scans were unavailable or unreadable^(42)^. Nonetheless, participants without complete AAC data were similar in baseline characteristics to those included in our study (online Supplementary Table S2). Baseline characteristics for all participants and by AAC score categories are presented in Table 1. There were 128/684 (18·7 %) who had extensive AAC and 495/684 (72·4 %) participants who had the presence of AAC.

**Table 1.** Baseline characteristics of all participants and by abdominal aortic calcification (AAC) score categories (Median values and interquartile ranges (IQR); numbers and percentages; mean values and standard deviations)

	All participants (*n* 684)			AAC score categories					
				AAC_24_ 0–5 (*n* 556)			AAC_24_ ≥ 6 (*n* 128)		
	Median		IQR	Median		IQR	Median		IQR
Age (years)									
Mean		74·9			74·8			75·5	
sd		2·6			2·6			2·7	
Ca treatment group									
*n*		350			287			63	
%		51·2			51·6			49·2	
Smoked ever									
*n*		230			173			57	
%		33·6			31·1			44·5	
Physical activity (kJ/d)	495·4		191·9–861·7	508·0		206·1–870·3	427·1		0·0–843·6
Dietary intakes									
ADG adherence score (0–60)									
Mean		29·4			29·6			28·4	
sd		8·2			8·1			8·6	
Total energy (kJ/d)									
Mean		7076·1			7107·9			6937·9	
sd		2055·6			1986·0			2337·6	
Total vegetables (g/d)	189·1		141·6–238·9	192·2		143·6–240·1	177·9		135·3–228·2
Cruciferous vegetables (g/d)	28·2		15·0–44·7	28·4		15·7–45·3	25·9		10·8–40·7
Leafy green vegetables (g/d)	17·3		10·1–25·9	17·3		9·9–25·9	16·8		10·4–26·6
Allium vegetables (g/d)	6·0		2·9–10·2	6·0		3·1–10·1	6·1		2·3–10·6
Yellow/orange/red vegetables (g/d)	49·4		33·6–68·2	50·3		33·8–68·4	45·7		32·3–67·3
Legumes (g/d)	22·4		13·7–34·1	23·0		13·7–34·3	20·6		13·7–32·5
BMI (kg/m^2^)									
Mean		26·9			27·0			26·2	
sd		4·3			4·4			3·8	
Anti-hypertensive medication									
*n*		257			201			56	
%		37·6			36·2			43·8	
Statin medication									
*n*		99			69			30	
%		14·5			12·4			23·4	
eGFR (ml/min per 1·73 m^2^)									
Mean		67·0			67·1			66·6	
sd		12·4			12·3			12·8	

ADG, Australian Dietary Guidelines; eGFR, estimated glomerular filtration rate.

### Associations of vegetable intakes with abdominal aortic calcification

Higher intake of cruciferous vegetables was associated with a lower odds of having extensive AAC (AAC24 ≥ 6) (Table 2). Every 20 g/d higher intake of cruciferous vegetables was associated with a 19 % lower odds of having extensive AAC after adjusting for lifestyle, dietary and cardiovascular risk factors (OR 0·81, 95 % CI 0·66, 0·99, *P* = 0·042). In quartile analyses, women in the highest quartile of cruciferous vegetable intake (>44·6 g/d) had a 46 % lower odds of having extensive AAC in comparison with women in the lowest quartile of cruciferous vegetable intake (<15·0 g/d; OR_Q4 *v.* Q1_ 0·54, 95 % CI 0·30, 0·97, *P* = 0·036). However, trivial evidence existed of a linear trend (*P*_trend_ = 0·072). No association was observed for total vegetables, leafy green vegetables, allium vegetables, yellow/orange/red vegetables and legumes with extensive AAC (*P* > 0·05 for all). Intake of total vegetables and individual types of vegetables was not related to the presence of AAC (AAC24 scores ≥1) (online Supplementary Table S3).

**Table 2.** Extensive abdominal aortic calcification 24 scores (AAC24 ≥ 6) by energy-adjusted intakes of total vegetables and vegetable types (Odds ratios and 95 % confidence intervals)

	OR (*n* 684)	95 % CI	*P*	Quartiles of vegetable types*							
				Q1	Q2		Q3		Q4		*P*_for trend_ †
					OR	95 % CI	OR	95 % CI	OR	95 % CI	
Total vegetables (75 g/d)											
Model 1	0·95	0·79, 1·14	0·576	Reference	0·67	0·40, 1·44	0·65	0·38, 1·10	0·62	0·36, 1·06	0·091
Model 2	1·01	0·82, 1·25	0·897	Reference	0·64	0·37, 1·10	0·64	0·37, 1·11	0·68	0·37, 1·23	0·218
Model 3	0·99	0·80, 1·22	0·916	Reference	0·62	0·36, 1·08	0·62	0·36, 1·09	0·66	0·36, 1·20	0·191
Cruciferous vegetables (20 g/d)											
Model 1	0·83	0·68, 1·00	0·055	Reference	0·61	0·36, 1·04	0·69	0·41, 1·17	0·55	0·32, 0·95	0·055
Model 2	0·82	0·67, 1·00	0·054	Reference	0·60	0·35, 1·04	0·74	0·43, 1·27	0·54	0·30, 0·96	0·072
Model 3	0·81	0·66, 0·99	0·042	Reference	0·60	0·34, 1·04	0·71	0·41, 1·23	0·54	0·30, 0·97	0·072
Leafy green vegetables (20 g/d)											
Model 1	1·13	0·80, 1·57	0·491	Reference	1·25	0·73, 2·15	0·95	0·54, 1·67	1·13	0·65, 1·96	0·893
Model 2	1·13	0·80, 1·57	0·491	Reference	1·33	0·77, 2·32	0·99	0·56, 1·77	1·25	0·71, 2·21	0·658
Model 3	1·14	0·81, 1·61	0·439	Reference	1·34	0·76, 2·35	1·05	0·59, 1·88	1·25	0·70, 2·22	0·654
Allium vegetables (20 g/d)											
Model 1	1·03	0·58, 1·84	0·919	Reference	0·68	0·39, 1·18	0·82	0·48, 1·40	0·87	0·52, 1·48	0·912
Model 2	1·10	0·58, 2·09	0·768	Reference	0·75	0·43, 1·31	0·83	0·48, 1·45	0·93	0·52, 1·67	0·996
Model 3	0·98	0·51, 1·87	0·955	Reference	0·74	0·42, 1·30	0·88	0·50, 1·54	0·89	0·49, 1·61	0·890
Yellow/orange/red vegetables (20 g/d)											
Model 1	0·99	0·86, 1·14	0·883	Reference	1·21	0·72, 2·03	0·70	0·39, 1·22	0·86	0·50, 1·49	0·312
Model 2	1·05	0·89, 1·23	0·555	Reference	1·34	0·78, 2·31	0·75	0·41, 1·36	1·00	0·54, 1·84	0·600
Model 3	1·03	0·88, 1·21	0·729	Reference	1·40	0·81, 2·42	0·72	0·39, 1·33	0·98	0·53, 1·83	0·535
Legumes (20 g/d)											
Model 1	1·03	0·84, 1·27	0·755	Reference	1·29	0·77, 2·16	0·76	0·43, 1·34	0·86	0·50, 1·49	0·309
Model 2	1·07	0·87, 1·33	0·528	Reference	1·31	0·77, 2·23	0·82	0·46, 1·46	0·94	0·53, 1·67	0·536
Model 3	1·06	0·85, 1·32	0·593	Reference	1·30	0·76, 2·24	0·82	0·46, 1·47	0·96	0·54, 1·73	0·604

eGFR, estimated glomerular filtration rate.

* Quartiles for total vegetables were Q1 (*n* 171; <141·5 g/d), Q2 (*n* 171; 141·5–188·9 g/d), Q3 (*n* 171; 189·0–238·9 g/d) and Q4 (*n* 171; >238·9 g/d); cruciferous vegetables were Q1 (*n* 171; <15·0 g/d), Q2 (*n* 174; 15·0–28·2 g/d), Q3 (*n* 168; 28·3–44·6 g/d) and Q4 (*n* 171; >44·6 g/d); leafy green vegetables were Q1 (*n* 171; <10·1 g/d), Q2 (*n* 171; 10·1–17·2 g/d), Q3 (*n* 172; 17·3–25·9 g/d) and Q4 (*n* 170; >25·9 g/d); allium vegetables were Q1 (*n* 172; <3·0 g/d), Q2 (*n* 172; 3·0–6·0 g/d), Q3 (*n* 169; 6·1–10·1 g/d) and Q4 (*n* 171; >10·1 g/d); yellow/orange/red vegetables were Q1 (*n* 172; <33·7 g/d), Q2 (*n* 170; 33·7–49·3 g/d), Q3 (*n* 171; 49·4–68·1 g/d) and Q4 (*n* 171; >68·1 g/d); and legumes were Q1 (*n* 172; <13·7 g/d), Q2 (*n* 171; 13·7–22·4 g/d), Q3 (*n* 170; 22·5–34·0 g/d) and Q4 (*n* 171; >34·0 g/d). Model 1: unadjusted. Model 2: age, the Calcium Intake Fracture Outcome Study treatment code, smoking status, physical activity, diet quality, energy intake and other vegetables (i.e. non-cruciferous vegetables when cruciferous vegetables were the exposure of interest). Model 3: model 2 plus BMI, use of antihypertensive medication, use of statin medication, and eGFR.

† *P*_for trend_ was obtained using the median value within each quartile group as a continuous variable.

### Additional analyses

There was a moderate positive correlation between intake of cruciferous vegetables and total vegetables (*ρ* = 0·52, *P* < 0·001). Negligible correlations existed between intake of cruciferous vegetables and leafy green vegetables (*ρ* = 0·15, *P* < 0·001), allium vegetables (*ρ* = 0·14, *P* < 0·001), yellow/orange/red vegetables (*ρ* = 0·17, *P* < 0·001) and legumes (*ρ* = 0·24, *P* < 0·001). Apple intake had a negligible positive correlation with cruciferous vegetable intake (*ρ* = 0·13, *P* < 0·001). Using model 3 with the additional adjustment for apple intake, those with high intakes of cruciferous vegetables (>44·6 g/d) had a 45 % lower odds of having extensive AAC (OR_Q4 *v.* Q1_ 0·55, 95 % CI 0·30, 0·99, *P* = 0·045) in comparison with those with low intakes (<15·0 g/d).

The association between cruciferous vegetables and extensive AAC was attenuated when we performed our stratification analysis by medication use. There were 56/257 (21·8 %) who had extensive AAC in those prescribed antihypertensive medications and 72/427 (16·9 %) in those not prescribed antihypertensive medications. For those with high intakes of cruciferous vegetables (>44·6 g/d) in comparison to those with low intakes (<15·0 g/d), the associations were attenuated in those prescribed antihypertensive medications (OR_Q4 *v.* Q1_ = 0·71, 95 % CI 0·28, 1·80, *P* = 0·467) and in those not prescribed antihypertensive medications (OR_Q4 *v.* Q1_ = 0·50, 95 % CI 0·23, 1·11, *P* = 0·087). However, the point estimates were trending similar. In those prescribed statin medications, there were 30/99 (30·3 %) who had extensive AAC and in those not prescribed statin medications, there were 98/487 (16·8 %). In those prescribed statin medications, the association and point estimate were substantially attenuated (OR_Q4 *v.* Q1_ = 1·57, 95 % CI 0·36, 6·84, *P* = 547). However, in those not prescribed statin medications, the association remained with a similar point estimate (OR_Q4 *v.* Q1_ = 0·45, 95 % CI 0·23, 0·88, *P* = 0·020). The aforementioned findings should be interpreted with caution as there may be insufficient power for performing subgroup analysis. A larger cohort would be required to adequately test such hypotheses.

## Discussion

We have demonstrated an association between cruciferous vegetable intakes and extensive AAC in older women. This is the first study to demonstrate a higher intake of cruciferous vegetables to be associated with lower odds for extensive AAC after adjustment for lifestyle, dietary and CVD risk factors. Since extensive AAC is strongly associated with a higher long-term risk of CVD hospitalisations and deaths^(5,43)^, our findings highlight the potential mechanisms by which cruciferous vegetables may play a role in reducing CVD risk. The stronger association observed in those not taking statin medication highlights the potential importance of diet especially among those not identified as requiring lipid-lowering medications.

Interestingly, when previously investigating the association of cruciferous vegetable intake with carotid artery disease^(13)^, we did not identify any association between intake of cruciferous vegetables and presence or severity of carotid atherosclerotic plaques. Rather, we observed that intake of cruciferous vegetables was inversely associated with common carotid artery intima-media thickness, a measure of thickening of the intima-media complex reflecting generalised atherosclerosis^(44)^. In this study, we found higher intake of cruciferous vegetables was associated with a lower odds of having extensive calcification of the abdominal aorta, an indicator of extensive blood vessel disease, suggesting that constituents of cruciferous vegetables may either affect the progression of atherosclerotic lesions or attenuate the pro-calcific processes often seen in older individuals.

Investigation into the dietary determinants of AAC is an emerging area of research. To date, most studies have evaluated relationships between dietary patterns and aortic calcification. While the significance of dietary patterns cannot be understated, investigation into the importance of specific dietary components is fundamental. Recently, Shang *et al.* examined the relationship between diet quality, assessed using the Alternative Healthy Eating Index-2010, and AAC in community-dwelling older adults (*n* 262)^(45)^. The authors reported that baseline, but not changes in Alternative Healthy Eating Index-2010, was inversely associated with extensive AAC. Higher Alternative Healthy Eating Index-2010 scores are reflective of a diet high in plant-based foods, such as vegetables, fruits, whole grains, nuts and legumes. These foods are also components of a Mediterranean-type diet, which has been linked with a lower degree and slower progression of coronary artery calcification^(46)^. An integral component of a healthy diet is a higher intake of vegetables and fruits, of which have been shown to be associated with a 26 % lower odds (95 % CI 0·56, 0·99) of having coronary artery calcification in a young adult population after 20 years of follow-up^(47)^.

Collectively, these findings may be explained by the numerous bioactive compounds found in vegetables and fruits. Vegetables and fruits provide a diverse range of bioactive compounds^(12,48)^. Specifically, these components may be involved in a number of protective mechanisms, such as the reduction of oxidative stress and inflammation, which are known to contribute to vascular calcification^(49,50)^. Previously, we reported a 24 % lower odds (95 % CI 0·62, 0·93) of having extensive AAC for every 50 g/d higher apple intake in the present cohort^(21)^. As we have now demonstrated that both cruciferous vegetables and apples are inversely associated with extensive AAC, it is possible that flavonols^(51)^, found abundantly in both of the aforementioned foods, may play a role. Furthermore, carbohydrate compounds, such as pectin found in both apples and cruciferous vegetables, may also contribute to the prevention of vascular calcification^(52)^. For example, supplementation of dietary pectin (15 g/d) over a 4 week period induced changes in plasma fibrin network characteristics^(53)^. These changes indicated network structures were more permeable with lower tensile strength, which are believed to be less atherogenic^(53)^. Other authors have also reported the possible role of Mg, which can be found in fruits and vegetables, on AAC^(54)^.

Phylloquinone, also known as vitamin K_1_, is another bioactive compound found abundantly in cruciferous vegetables that could partially explain the inverse association we have observed with AAC. Phylloquinone is the most common form of vitamin K compounds and is mainly found in leafy green vegetables, broccoli and Brussels sprouts^(55)^. The other major vitamin K compounds are a group of bacterial menaquinones (or vitamin K_2_). Menaquinones regulate vitamin K-dependent proteins, such as matrix Gla protein, of which inhibit vascular calcification^(56)^. Although menaquinones occur primarily in animal-based foods, there is evidence that phylloquinone can break down to menadione, an intermediate which is then converted to menoquinone-4^(57)^; therefore, potentially inhibiting vascular calcification.

Limitations must be considered when interpreting the findings from this study. Due to the observational nature and cross-sectional design of this study, causality cannot be established. We cannot rule out residual confounding, other factors associated with a healthy lifestyle, or the possibility of reverse causality bias. However, reverse causality bias is unlikely due to the asymptomatic nature of aortic calcification. Furthermore, we cannot rule out the possibility of selection bias affecting our results as the entire cohort was recruited on the basis of being ambulant and the likelihood of surviving beyond 5 years and we did not have complete AAC data as some scans were unavailable or unreadable^(42)^. Nonetheless, participants without complete AAC data were similar in baseline characteristics to those included in our study. In addition to the above limitations, we cannot rule out the possibility of measurement error, provided that it was non-differential, in the assessment of our dietary data. However, measurement error would likely lead to an underestimation of an association and a reduction in power for detecting an association^(58)^. Hence, our finding that cruciferous vegetable intake is inversely associated with extensive AAC is robust to this limitation. We also have confidence the FFQ used in our study gives a robust estimate for total vegetables and the classified types of vegetables due to the reasonably good agreement for *β*-carotene, fibre and vitamin C (*r* 0·43, 0·66 and 0·52, respectively) between weighed food records and the FFQ used in our study^(24)^. Lastly, these findings cannot be extended to men, younger women or ethnicities that are not Caucasian. Further studies are needed to confirm these findings in these populations.

There were several strengths to our study. We had detailed information of lifestyle and CVD risk factors, medications and disease history. Second, glomerular filtration rate was estimated using the combined creatinine–cystatin C equation, which performs better than equations based on creatinine alone^(39)^. This is an important consideration as poor renal function is linked to higher calcification of the vasculature^(59)^. Lastly, AAC was assessed by a single highly experienced investigator (J. T. S.) blinded to the clinical data from the study with both intra- and inter-rater agreements by J. T. S. being reported as very good^(7,20)^.

Overall, this study adds strength to the conceptual framework that cruciferous vegetables may be protective against particular aspects of structural vascular disease affecting vessel wall properties as we have now demonstrated an inverse association with these measures at two different locations in the arterial tree (common carotid artery^(13)^ and abdominal aorta) as well as a lower risk of atherosclerotic vascular disease and all-cause mortality^(14)^. Although these findings are only hypothesis generating and cannot imply causality, they suggest new avenues to explore the cardiovascular health benefits of bioactive constituents of cruciferous vegetables. Further studies of similar design are needed to confirm these findings in male and younger female cohorts and non-Caucasian populations. Large, long-term randomised controlled trials are also needed to support causality as well as investigations to determine the biochemical pathways involved.

## Supplementary material

For supplementary material referred to in this article, please visit https://doi.org/10.1017/S0007114520002706
